# 2-Pyridone: monoclinic polymorph

**DOI:** 10.1107/S1600536809049496

**Published:** 2009-11-25

**Authors:** Hadi D. Arman, Pavel Poplaukhin, Edward R. T. Tiekink

**Affiliations:** a Department of Chemistry, The University of Texas at San Antonio, One UTSA Circle, San Antonio, Texas 78249-0698, USA; b Chemical Abstracts Service, 2540 Olentangy River Rd, Columbus, Ohio 43202, USA; c Department of Chemistry, University of Malaya, 50603 Kuala Lumpur, Malaysia

## Abstract

The asymmetric unit in the title compound, C_5_H_5_NO, comprises two independent but virtually identical mol­ecules of 2-pyridone, and represents a monoclinic polymorph of the previously reported ortho­rhom­bic (*P*2_1_2_1_2_1_) form [Penfold (1953 ▶). *Acta Cryst.* **6**, 591–600; Ohms *et al.* (1984 ▶). *Z. Kristallogr.* **169**, 185–200; Yang & Craven (1998 ▶). *Acta Cryst.* B**54**, 912–920]. The independent mol­ecules are linked into supra­molecular dimers *via* eight-membered {⋯HNC(O)}_2_ amide synthons in contrast to the helical supra­molecular chains, mediated by {⋯HNC(O)} links, found in the ortho­rhom­bic form.

## Related literature

For the structure of the ortho­rhom­bic form of 2-pyridone, see: Penfold (1953 ▶); Ohms *et al.* (1984 ▶); Yang & Craven (1998 ▶). For related studies of co-crystal formation, see: Broker & Tiekink (2007 ▶); Ellis *et al.* (2009 ▶). For analysis of the geometric structures, see: Spek (2009 ▶).
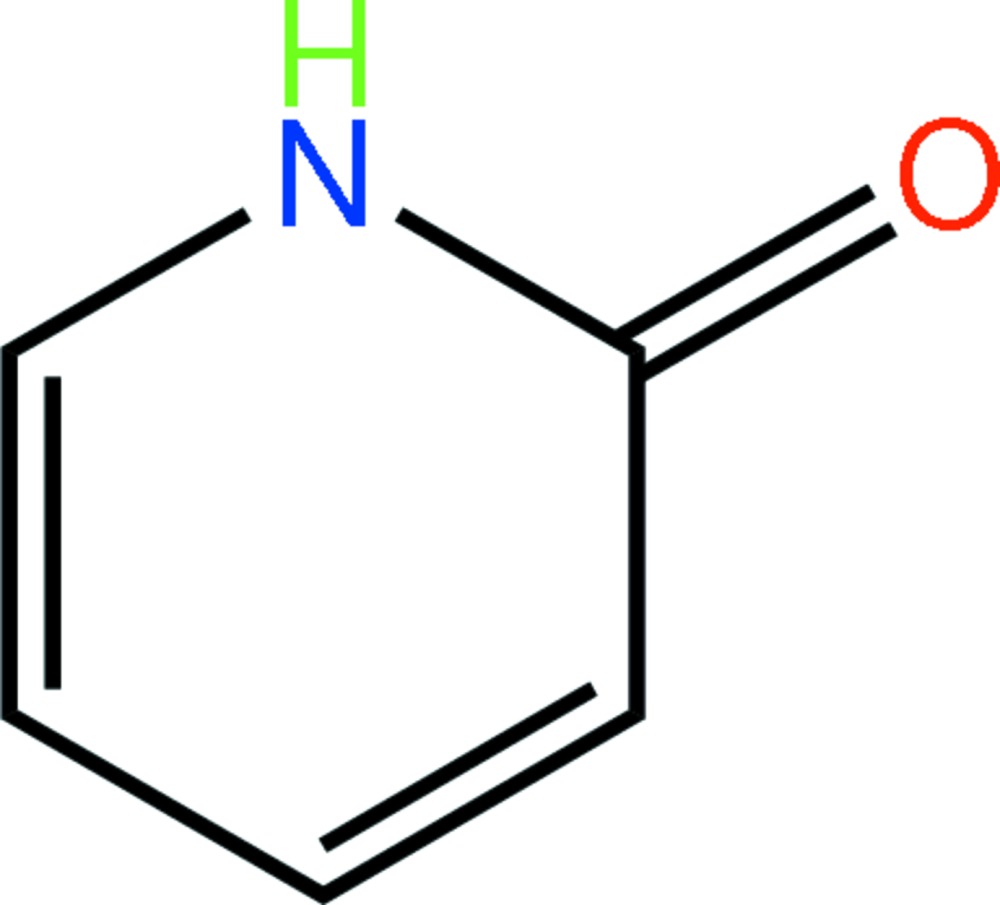


## Experimental

### 

#### Crystal data

C_5_H_5_NO
*M* *_r_* = 95.10
Monoclinic, 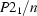

*a* = 6.2027 (13) Å
*b* = 16.327 (4) Å
*c* = 9.1046 (18) Å
β = 92.242 (7)°
*V* = 921.3 (3) Å^3^
*Z* = 8
Mo *K*α radiation
μ = 0.10 mm^−1^
*T* = 98 K
0.44 × 0.39 × 0.15 mm

#### Data collection

Rigaku AFC12K/SATURN724 diffractometer
Absorption correction: multi-scan (*ABSCOR*; Higashi, 1995 ▶) *T*_min_ = 0.840, *T*_max_ = 1
6582 measured reflections
1903 independent reflections
1724 reflections with *I* > 2σ(*I*)
*R*_int_ = 0.037

#### Refinement

*R*[*F* ^2^ > 2σ(*F* ^2^)] = 0.044
*wR*(*F* ^2^) = 0.117
*S* = 1.10
1903 reflections
127 parameters
H-atom parameters constrained
Δρ_max_ = 0.21 e Å^−3^
Δρ_min_ = −0.22 e Å^−3^

### 

Data collection: *CrystalClear* (Rigaku/MSC, 2005 ▶); cell refinement: *CrystalClear*; data reduction: *CrystalClear*; program(s) used to solve structure: *SHELXS97* (Sheldrick, 2008 ▶); program(s) used to refine structure: *SHELXL97* (Sheldrick, 2008 ▶); molecular graphics: *DIAMOND* (Brandenburg, 2006 ▶); software used to prepare material for publication: *publCIF* (Westrip, 2009 ▶).

## Supplementary Material

Crystal structure: contains datablocks global, I. DOI: 10.1107/S1600536809049496/hg2602sup1.cif

Structure factors: contains datablocks I. DOI: 10.1107/S1600536809049496/hg2602Isup2.hkl

Additional supplementary materials:  crystallographic information; 3D view; checkCIF report

## Figures and Tables

**Table 1:** Hydrogen-bond geometry (Å, °)

*D*—H⋯*A*	*D*—H	H⋯*A*	*D*⋯*A*	*D*—H⋯*A*
N1—H1n⋯O2	0.88	1.86	2.7450 (16)	177
N2—H2n⋯O1	0.88	1.92	2.7915 (16)	171
C2—H2⋯O1^i^	0.95	2.53	3.3943 (18)	150
C4—H4⋯O2^ii^	0.95	2.54	3.2989 (18)	137

Symmetry codes: (i) 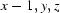; (ii) 
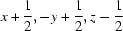
.

**Fig. 1.**
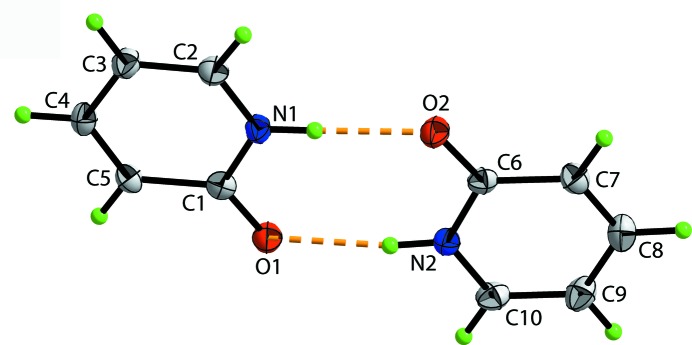
Molecular structures of the two independent molecules comprising the asymmetric unit in (I), showing atom-labelling scheme and displacement ellipsoids at the 50% probability level. The molecules are connected by N–H···O hydrogen bonds (orange dashed lines).

**Fig. 2.**
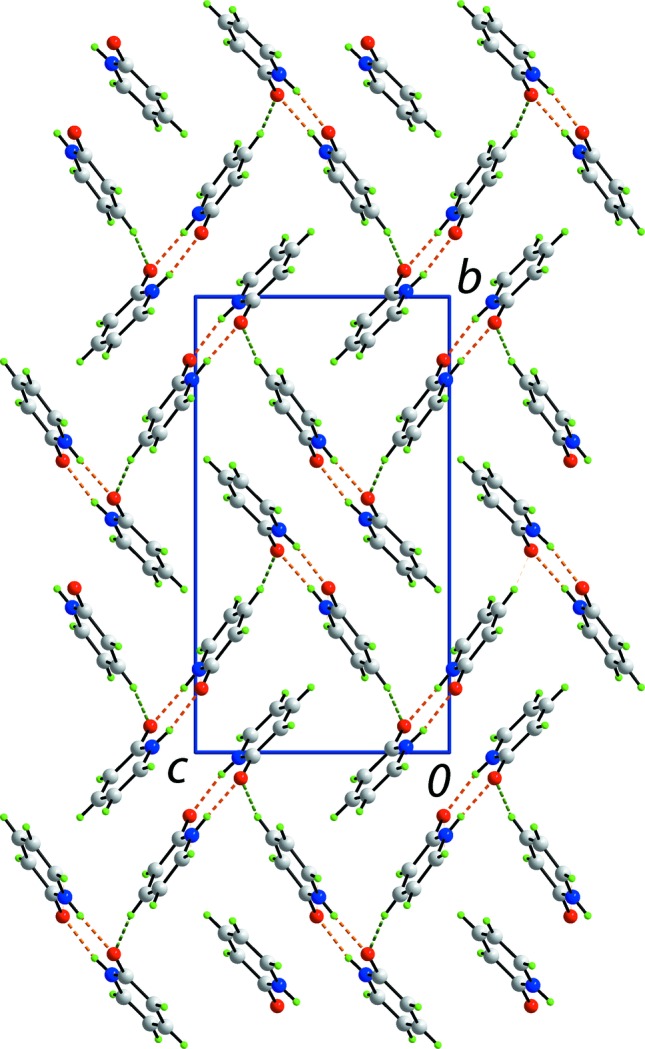
View of the stacking of layers along the b axis in crystal structure of (I). Colour code: O, red; N, blue; C, grey; and H, green. The N–H···O hydrogen bonds (orange) and C–H···O contacts (green) are shown as dashed lines.

## supplementary crystallographic information

### Comment

Crystals of the monoclinic polymorph of 2-pyridone, (I), were isolated during an on-going study into the phenomenon of co-crystal formation (Broker & Tiekink, 2007; Ellis *et al.*, 2009). The orthorhombic form of (I) has been characterized previously (Penfold, 1953; Ohms *et al.*, 1984; Yang & Craven, 1998).

In (I), two independent molecules comprise the asymmetric unit, Fig. 1, and these are virtually identical as seen in the r.m.s. values for bond distances and angles of 0.0025 Å and 0.184 °, respectively (Spek, 2009). Each molecule is essentially planar with the maximum deviation of 0.0102 (14) Å found for the C2 atom in the N1-molecule and 0.0029 (14) Å for the C6 atom in the N2-molecule. The pattern of bond distances matches those in the previously determined orthorhombic form.

The crystal packing in (I) is sustained by eight-membered {···HNC(O)}_2_ amide synthons whereby the two independent molecules are linked, Table 1 and Fig. 1. The dimeric aggregate is effectively planar with the dihedral between the two 2-pyridone rings being 7.88 (6) °, The dimers are connected into zigzag layers in the *ac* plane *via* C—H···O interactions, Table 1 and Fig. 2. The major difference between the two polymeric forms of 2-pyridone rests in the mode of association between the 2-pyridone molecules. In the orthorhombic form, the molecules are lined into supramolecular helical chains through a continuing sequence of {···HNC( O)} links.

### Experimental

2-Hydroxypyridine (Fluka) was dissolved in chloroform and layered with hexanes. Large rod-like colourless crystals formed within a week.

### Refinement

The N– and C-bound H-atoms were placed in calculated positions (N–H = 0.88 Å and C–H 0.95 Å) and were included in the refinement in the riding model approximation with *U*_iso_(H) set to 1.2*U*_eq_(N, C).

### Crystal data

**Table:**

C_5_H_5_NO	*F*(000) = 400
*M**_r_* = 95.10	*D*_x_ = 1.371 Mg m^−^^3^
Monoclinic, *P*2_1_/*n*	Mo *K*α radiation, λ = 0.71069 Å
Hall symbol: -P 2yn	Cell parameters from 3046 reflections
*a* = 6.2027 (13) Å	θ = 3.3–40.2°
*b* = 16.327 (4) Å	µ = 0.10 mm^−^^1^
*c* = 9.1046 (18) Å	*T* = 98 K
β = 92.242 (7)°	Prism, colourless
*V* = 921.3 (3) Å^3^	0.44 × 0.39 × 0.15 mm
*Z* = 8	

### Data collection

**Table:**

Rigaku AFC12K/SATURN724 diffractometer	1903 independent reflections
Radiation source: fine-focus sealed tube	1724 reflections with *I* > 2σ(*I*)
graphite	*R*_int_ = 0.037
ω scans	θ_max_ = 26.5°, θ_min_ = 2.5°
Absorption correction: multi-scan (*ABSCOR*; Higashi, 1995)	*h* = −7→5
*T*_min_ = 0.840, *T*_max_ = 1	*k* = −20→20
6582 measured reflections	*l* = −11→11

### Refinement

**Table:**

Refinement on *F*^2^	Primary atom site location: structure-invariant direct methods
Least-squares matrix: full	Secondary atom site location: difference Fourier map
*R*[*F*^2^ > 2σ(*F*^2^)] = 0.044	Hydrogen site location: inferred from neighbouring sites
*wR*(*F*^2^) = 0.117	H-atom parameters constrained
*S* = 1.10	*w* = 1/[σ^2^(*F*_o_^2^) + (0.0583*P*)^2^ + 0.275*P*] where *P* = (*F*_o_^2^ + 2*F*_c_^2^)/3
1903 reflections	(Δ/σ)_max_ = 0.001
127 parameters	Δρ_max_ = 0.21 e Å^−^^3^
0 restraints	Δρ_min_ = −0.22 e Å^−^^3^

### Special details

**Table:**

Geometry. All s.u.'s (except the s.u. in the dihedral angle between two l.s. planes) are estimated using the full covariance matrix. The cell s.u.'s are taken into account individually in the estimation of s.u.'s in distances, angles and torsion angles; correlations between s.u.'s in cell parameters are only used when they are defined by crystal symmetry. An approximate (isotropic) treatment of cell s.u.'s is used for estimating s.u.'s involving l.s. planes.
Refinement. Refinement of *F*^2^ against ALL reflections. The weighted *R*-factor *wR* and goodness of fit *S* are based on *F*^2^, conventional *R*-factors *R* are based on *F*, with *F* set to zero for negative *F*^2^. The threshold expression of *F*^2^ > 2σ(*F*^2^) is used only for calculating *R*-factors(gt) *etc*. and is not relevant to the choice of reflections for refinement. *R*-factors based on *F*^2^ are statistically about twice as large as those based on *F*, and *R*- factors based on ALL data will be even larger.

### Fractional atomic coordinates and isotropic or equivalent isotropic displacement parameters (Å^2^)

**Table:**

	*x*	*y*	*z*	*U*_iso_*/*U*_eq_	
O1	0.27541 (14)	0.36020 (6)	0.47704 (10)	0.0239 (2)	
N1	−0.07408 (17)	0.31779 (7)	0.48531 (12)	0.0201 (3)	
H1N	−0.1007	0.3587	0.5450	0.024*	
C1	0.1321 (2)	0.30993 (8)	0.43716 (14)	0.0195 (3)	
C2	−0.2397 (2)	0.26660 (8)	0.44686 (15)	0.0232 (3)	
H2	−0.3795	0.2769	0.4819	0.028*	
C3	−0.2074 (2)	0.20065 (8)	0.35857 (15)	0.0236 (3)	
H3	−0.3219	0.1641	0.3330	0.028*	
C4	0.0019 (2)	0.18832 (8)	0.30615 (14)	0.0224 (3)	
H4	0.0289	0.1426	0.2450	0.027*	
C5	0.1649 (2)	0.24113 (8)	0.34226 (14)	0.0209 (3)	
H5	0.3033	0.2324	0.3039	0.025*	
O2	−0.14759 (15)	0.44304 (6)	0.67815 (11)	0.0263 (3)	
N2	0.19925 (17)	0.48837 (7)	0.67120 (12)	0.0204 (3)	
H2N	0.2238	0.4519	0.6027	0.024*	
C6	−0.0049 (2)	0.49182 (8)	0.72495 (14)	0.0199 (3)	
C7	−0.0344 (2)	0.55309 (8)	0.83511 (15)	0.0236 (3)	
H7	−0.1717	0.5592	0.8765	0.028*	
C8	0.1311 (2)	0.60263 (9)	0.88132 (16)	0.0267 (3)	
H8	0.1078	0.6427	0.9547	0.032*	
C9	0.3370 (2)	0.59536 (9)	0.82168 (17)	0.0276 (3)	
H9	0.4526	0.6299	0.8539	0.033*	
C10	0.3652 (2)	0.53746 (9)	0.71679 (15)	0.0243 (3)	
H10	0.5022	0.5313	0.6751	0.029*	

### Atomic displacement parameters (Å^2^)

**Table:**

	*U*^11^	*U*^22^	*U*^33^	*U*^12^	*U*^13^	*U*^23^
O1	0.0186 (5)	0.0256 (5)	0.0277 (5)	−0.0021 (4)	0.0025 (4)	−0.0048 (4)
N1	0.0186 (6)	0.0198 (5)	0.0219 (5)	0.0010 (4)	0.0027 (4)	−0.0035 (4)
C1	0.0180 (6)	0.0210 (6)	0.0195 (6)	0.0012 (5)	0.0000 (5)	0.0018 (5)
C2	0.0178 (6)	0.0248 (7)	0.0272 (7)	−0.0013 (5)	0.0026 (5)	−0.0022 (5)
C3	0.0208 (7)	0.0220 (7)	0.0281 (7)	−0.0021 (5)	0.0013 (5)	−0.0036 (5)
C4	0.0244 (7)	0.0202 (6)	0.0227 (6)	0.0036 (5)	−0.0004 (5)	−0.0025 (5)
C5	0.0179 (6)	0.0239 (7)	0.0211 (6)	0.0031 (5)	0.0031 (5)	−0.0010 (5)
O2	0.0206 (5)	0.0245 (5)	0.0342 (5)	−0.0041 (4)	0.0062 (4)	−0.0078 (4)
N2	0.0209 (6)	0.0183 (5)	0.0221 (5)	−0.0003 (4)	0.0029 (4)	−0.0014 (4)
C6	0.0191 (6)	0.0183 (6)	0.0222 (6)	0.0011 (5)	0.0009 (5)	0.0029 (5)
C7	0.0212 (6)	0.0252 (7)	0.0245 (7)	0.0031 (5)	0.0025 (5)	−0.0007 (5)
C8	0.0268 (7)	0.0262 (7)	0.0270 (7)	0.0035 (5)	−0.0010 (6)	−0.0063 (5)
C9	0.0227 (7)	0.0256 (7)	0.0342 (8)	−0.0026 (5)	−0.0024 (6)	−0.0043 (6)
C10	0.0176 (6)	0.0251 (7)	0.0304 (7)	−0.0016 (5)	0.0015 (5)	0.0016 (6)

### Geometric parameters (Å, °)

**Table:**

O1—C1	1.2529 (16)	O2—C6	1.2530 (16)
N1—C2	1.3597 (17)	N2—C10	1.3567 (17)
N1—C1	1.3743 (17)	N2—C6	1.3762 (17)
N1—H1N	0.8800	N2—H2N	0.8800
C1—C5	1.4365 (18)	C6—C7	1.4335 (18)
C2—C3	1.3633 (19)	C7—C8	1.3607 (19)
C2—H2	0.9500	C7—H7	0.9500
C3—C4	1.4151 (18)	C8—C9	1.412 (2)
C3—H3	0.9500	C8—H8	0.9500
C4—C5	1.3590 (19)	C9—C10	1.360 (2)
C4—H4	0.9500	C9—H9	0.9500
C5—H5	0.9500	C10—H10	0.9500
			
C2—N1—C1	124.33 (11)	C10—N2—C6	124.37 (11)
C2—N1—H1N	117.8	C10—N2—H2N	117.8
C1—N1—H1N	117.8	C6—N2—H2N	117.8
O1—C1—N1	120.32 (11)	O2—C6—N2	120.04 (12)
O1—C1—C5	124.83 (12)	O2—C6—C7	124.99 (12)
N1—C1—C5	114.84 (11)	N2—C6—C7	114.96 (11)
N1—C2—C3	120.67 (12)	C8—C7—C6	121.03 (12)
N1—C2—H2	119.7	C8—C7—H7	119.5
C3—C2—H2	119.7	C6—C7—H7	119.5
C2—C3—C4	118.00 (12)	C7—C8—C9	120.93 (13)
C2—C3—H3	121.0	C7—C8—H8	119.5
C4—C3—H3	121.0	C9—C8—H8	119.5
C5—C4—C3	120.77 (12)	C10—C9—C8	118.12 (13)
C5—C4—H4	119.6	C10—C9—H9	120.9
C3—C4—H4	119.6	C8—C9—H9	120.9
C4—C5—C1	121.36 (12)	N2—C10—C9	120.59 (12)
C4—C5—H5	119.3	N2—C10—H10	119.7
C1—C5—H5	119.3	C9—C10—H10	119.7
			
C2—N1—C1—O1	179.47 (12)	C10—N2—C6—O2	−178.77 (12)
C2—N1—C1—C5	−1.04 (18)	C10—N2—C6—C7	0.61 (18)
C1—N1—C2—C3	2.1 (2)	O2—C6—C7—C8	178.83 (13)
N1—C2—C3—C4	−1.3 (2)	N2—C6—C7—C8	−0.51 (19)
C2—C3—C4—C5	−0.4 (2)	C6—C7—C8—C9	0.2 (2)
C3—C4—C5—C1	1.5 (2)	C7—C8—C9—C10	0.0 (2)
O1—C1—C5—C4	178.72 (12)	C6—N2—C10—C9	−0.4 (2)
N1—C1—C5—C4	−0.74 (18)	C8—C9—C10—N2	0.1 (2)

### Hydrogen-bond geometry (Å, °)

**Table:**

*D*—H···*A*	*D*—H	H···*A*	*D*···*A*	*D*—H···*A*
N1—H1n···O2	0.88	1.86	2.7450 (16)	177
N2—H2n···O1	0.88	1.92	2.7915 (16)	171
C2—H2···O1^i^	0.95	2.53	3.3943 (18)	150
C4—H4···O2^ii^	0.95	2.54	3.2989 (18)	137

Symmetry codes: (i) *x*−1, *y*, *z*; (ii) *x*+1/2, −*y*+1/2, *z*−1/2.
